# The Vertebrate Breed Ontology: Towards Effective Breed Data Standardization

**Published:** 2024-06-03

**Authors:** Kathleen R. Mullen, Imke Tammen, Nicolas A. Matentzoglu, Marius Mather, Christopher J. Mungall, Melissa A. Haendel, Frank W. Nicholas, Sabrina Toro, Vertebrate Breed Ontology Consortium

**Affiliations:** 1 University of Colorado Anschutz Medical Campus; 2 University of Sydney; 3 Semanticly Ltd; 4 Lawrence Berkeley National Laboratory; 5 University of North Carolina at Chapel Hill

## Abstract

**Background –:**

Limited universally adopted data standards in veterinary science hinders data interoperability and therefore integration and comparison; this ultimately impedes application of existing information-based tools to support advancement in veterinary diagnostics, treatments, and precision medicine.

**Hypothesis/Objectives –:**

Creation of a Vertebrate Breed Ontology (VBO) as a single, coherent logic-based standard for documenting breed names in animal health, production and research-related records will improve data use capabilities in veterinary and comparative medicine.

**Animals –:**

No live animals were used in this study.

**Methods –:**

A list of breed names and related information was compiled from relevant sources, organizations, communities, and experts using manual and computational approaches to create VBO. Each breed is represented by a VBO term that includes all provenance and the breed’s related information as metadata. VBO terms are classified using description logic to allow computational applications and Artificial Intelligence-readiness.

**Results –:**

VBO is an open, community-driven ontology representing over 19,000 livestock and companion animal breeds covering 41 species. Breeds are classified based on community and expert conventions (e.g., horse breed, cattle breed). This classification is supported by relations to the breeds’ genus and species indicated by NCBI Taxonomy terms. Relationships between VBO terms, e.g. relating breeds to their foundation stock, provide additional context to support advanced data analytics. VBO term metadata includes common names and synonyms, breed identifiers/codes, and attributed cross-references to other databases.

**Conclusion and clinical importance –:**

Veterinary data interoperability and computability can be enhanced by the adoption of VBO as a source of standard breed names in databases and veterinary electronic health records.

## Introduction

Precision veterinary medicine holds great promise for advancing disease characterization and targeted drug therapies for animals, as exemplified in the fields of veterinary genomics and oncology.^1–7^ The success of individualized patient care relies on the availability of data, including molecular mechanisms of disease, genomic profiling, and pharmacogenomics,^3,6^ available from research databases and health records. However, non-human animal and veterinary data are rarely represented in databases and electronic health records (EHR) using universally accepted standards, making computability and integration of these data challenging. Standards are critical for data integration, as they ensure that data is comparable and consistent across sources by referring to similar concepts. For example, by using an NCBI gene identifier (ID) to represent a “gene”, a data entry is unambiguous, and related information referring to the same ID can confidently be combined and compared. Standard terminologies and data models have been widely accepted in model organism and human databases and EHR, fostering data interoperability, integration, and comparison; therefore supporting precision medicine applications. Ontologies have been accepted as gold standard terminologies: not only do ontology terms represent clearly defined concepts including synonyms and other rich metadata, but also, ontologies include computable relationships between terms within the same ontology and across resources, providing increased context and interoperability with a broad range of data for use during analytics.^8^

Breed name information is often embedded in free text notes, or flat lists in, for example, in practice management software. These lists vary between data sources and rarely connect with each other (e.g. there is rarely any indication that “German Shepherd”, “German Shepherd Dog”, “Alsatian”, and “Deutscher Schäferhund” refer to the same dog breed concept). Some breed name standards exist, e.g., Systematized Nomenclature of Medicine – Clinical Terms (SNOMED CT), The Livestock Breed Ontology (LBO, https://www.animalgenome.org/bioinfo/projects/lbo/), and the breed-name component of Veterinary Nomenclature (VeNom) Codes (https://venomcoding.org/). However, these standards are either limited in scope (e.g. LBO is limited to livestock breeds), impose restricted licenses, or lack an ontological foundation, making these existing standards unsuitable for a wide range of uses from animal husbandry to companion animal veterinary medicine data. An open-source standard that reconciles breed names, supporting information, and their provenance is needed globally to ensure data interoperability needed for precision medicine, learning healthcare, and to inform care guidelines and breeding best practices.

Here we introduce the Vertebrate Breed Ontology (VBO) as an open, comprehensive source for breed names and metadata across all vertebrate animals, including livestock and companion animals. VBO provides linkouts to resources and supporting information, and is actively maintained and continually enhanced, providing a powerful tool for breed-related data interoperability. We describe the process of creating VBO, its maintenance by the community, and how the use of VBO can support breed-information data interoperability, integration, and precision veterinary medicine.

## Materials and Methods

### Sources from breeds and related information:

Through active, collaborative engagements with international organizations, communities, and experts, we gathered lists of breeds and related breed information from relevant sources. A full list of these sources can be found in the VBO documentation.^9^ Each breed source has specific goals; for example, the Food and Agricultural Organization (FAO) Domestic Animal Diversity Information System (DAD-IS)^10^ aims to support breed conservation around the world, while canine and feline registration bodies focus on breed standards and documentation. Information included in breed lists is specific to the sources and can be contradictory. We included all information without discrimination and ensured provenance and attribution to these sources, as well as any decisions required to mitigate conflicts or discrepancies.

### Creation of the VBO content

We manually reviewed and curated the breed lists to (1) group information related to the same breed under the same VBO term, (2) create VBO term names based on the most commonly used breed name and species, ensuring term label uniqueness (see VBO documentation^9^), and (3) map breeds to their corresponding NCBI Taxonomy (NCBITaxon) record (representing the species). VBO terms were integrated within the NCBITaxon hierarchy using the *is_a* relation. Relations between breeds (e.g. indicating the breed’s foundation stock) were also manually curated based on breed information gathered by sources.

To facilitate ontology browsing and use, we created high-level grouping terms such as ‘dog breed’, ‘cattle breed’,… that were logically defined based on their NCBITaxon parentage (species, genus, or family). A description logic reasoner was leveraged to automatically classify VBO terms under these high-level terms. Rich metadata and cross-references to other terminologies and databases, including their provenance, were recorded for each VBO term.

### Creation of VBO using the Ontology Development Kit (ODK)

The Ontology Development Kit (ODK)^11^ provides a framework for creating ontologies, including both executable workflows for managing ontologies, such as release workflow and continuous integration, as well as ontology-processing tools such as ROBOT.^12^ The ODK is used to automatically check the VBO for errors whenever changes are proposed (e.g. new classes are added), and to release new ontology versions. VBO is managed and openly available on GitHub at https://github.com/monarch-initiative/vertebrate-breed-ontology. VBO has been accepted into the Open Biological and Biomedical Ontology (OBO) Foundry (https://obofoundry.org/).^13^

### VBO maintenance

Most external breed sources do not have unique and permanent identifiers that allow for a robust automated workflow. Therefore, VBO is currently mostly maintained based on user reviews and requests for changes or additions of new breeds submitted to the VBO GitHub repository (https://github.com/monarch-initiative/vertebrate-breed-ontology/issues).

More information about ontology content and maintenance can be found in Table 1 including minimum information for the reporting of an ontology (MIRO).^14^

## Results

### VBO as a standard for breeds and breed information

VBO was created as a single source for vertebrate animal breeds and related information. Since the concept of “breed” is not clearly defined between communities, we took a broad approach to defining ‘vertebrate breed’: “a group of animals that share specific characteristics (such as traits, behavior, genetics) that distinguish it from other organisms of the same species, and/or for which cultural or geographical separation has led to the general acceptance of its separate identity. Breeds are formed through genetic isolation and either natural adaptation to the environment or selective breeding, or a combination of the two.” We created breed concepts/VBO terms when they were officially recognized by an international breed organization, or when other groups or communities identify groups of animals as a breed.

**Figure 1:**
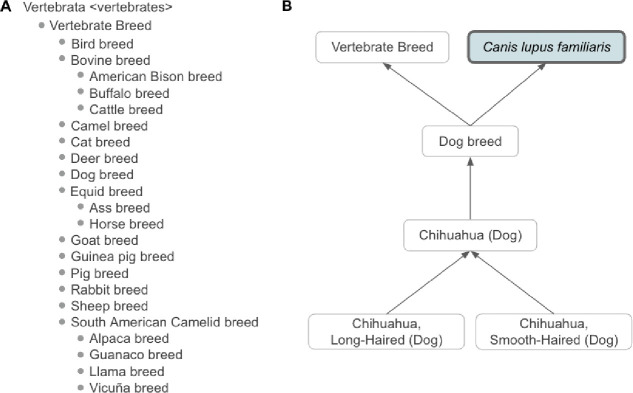
Classification of vertebrate breeds in VBO. **(A)** High-level classification based on species (e.g. ‘Dog breed’) and community usage (e.g. ‘Cattle breed”). **(B)** VBO representation of Chihuahua dog breeds in VBO. ‘Chihuahua (dog)’ is a subclass of ‘Dog breed’, itself a subclass of ‘Vertebrate Breed’ and ‘Canis lupus familiaris’. ‘Chihuahua, Long-Haired (Dog)’ and ‘Chihuahua, Smooth-Haired (Dog)’ are subclasses of ‘Chihuahua (dog)’, since they are more specific instances of ‘Chihuahua (dog)’. Term from NCBITaxon hierarchy is shown in thick border box. Arrows represent is_a relation. Some relations and VBO terms are not displayed in this figure for clarity. All VBO and NCBITaxon IDs are reported in Table 2.

**Figure 2:**
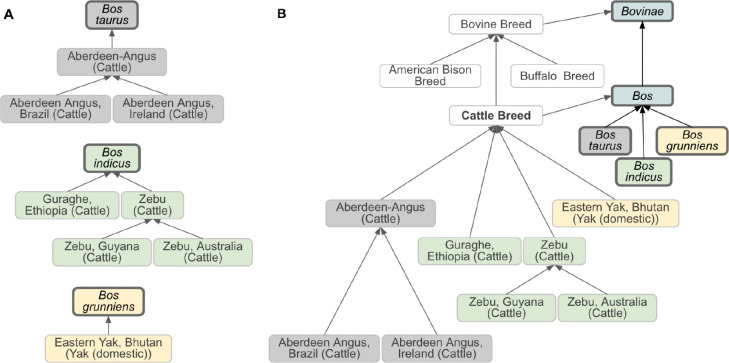
Classification of bovine breeds. **(A)** Relation between selected cattle breeds and their NCBITaxon species of *Bos taurus, Bos indicus, and Bos grunniens*. Each VBO term is related to an NCBITaxon. Breeds defined as having been reported in a specific country by National Coordinators in DAD-IS are either direct subclasses of their corresponding NCBITaxon or subclasses of other breeds (and therefore inherit the NCBITaxon subclass). Direct subclasses on NCBITaxon shown in this figure are ‘Guraghe, Ethiopia (Cattle)’ and ‘Eastern Yak, Bhutan (Yak (domestic))’. Subclasses of other breeds shown in this figure are: ‘Aberdeen Angus, Brazil (Cattle)’ and ‘Aberdeen Angus, Ireland (Cattle)’, subclasses of ‘Aberdeen-Angus (Cattle)’, and ‘Zebu, Guyana (Cattle)’ and ‘Zebu, Australia (Cattle)’, subclasses of ‘Zebu (Cattle)’. **(B)** ‘Cattle Breed’ is defined as “Vertebrate breed of the taxon *Bos*”, and is, therefore, a subclass of *Bos*. *Bos* encompasses *Bos taurus, Bos indicus, and Bos grunniens.* As a consequence, all breeds of these NCBITaxon species (as shown in A) are classified under ‘Cattle Breed’. Similarly, ‘Bovine Breed’ being defined as “Vertebrate breed of the taxon *Bovinae”* encompasses ‘Cattle Breed’ (of taxon *Bos)*, ‘American Bison Breed’ (of taxon *Bison bison)*, and ‘Buffalo Breed’ (of taxon *Bubalus bubalis), s*ince *Bos*, *Bison bison* and *Bubalus bubalis* are subclasses of *Bovinae* (not shown). Terms from NCBITaxon hierarchy are shown in thick border boxes. Arrows represent *is_a* relation. Some relations and VBO terms are not displayed in this figure for clarity. All VBO and NCBITaxon IDs are reported in Table 2.

VBO is an OBO Foundry ontology and is openly available and browsable in ontology browsers such as Ontobee (https://ontobee.org/ontology/VBO), the EMBL-EBI Ontology Lookup Service (OLS, https://www.ebi.ac.uk/ols4/ontologies/vbo), and BioPortal (https://bioportal.bioontology.org/ontologies/VBO). The v2024–05-24 version of VBO includes 19,121 terms representing breeds from vertebrate species, such as livestock (e.g. horse, cattle, chicken) and companion animals (e.g. dogs, cats), covering 41 species. VBO breed terms are classified under the general term ‘Vertebrate breed’ (VBO:0400000, Figure 1A), and grouped based on specific animal species (e.g. ‘Dog breed’, Figure 1B) or group of animals of the same genus (‘Cattle breed’, Figure 2) based on community and expert usage and jargon.

For example, ‘Dog breed’ (VBO:0400024) is defined at the species level as a “vertebrate breed of the taxon *Canis lupus familiaris*.” This definition excludes wild species such as *Canis rufus* (red wolf), *Canis latrans* (coyote) and *Canis lupus* (gray wolf). In contrast, ‘Cattle breed’ (VBO:0400020) is named based on the name used in agriculture, and defined at the genus level as a “vertebrate breed of the taxon *Bos*.” ‘Cattle breed’ groups classes for all breeds of *Bos* (NCBITaxon:9903) including *Bos indicus* (zebu cattle, NCBITaxon:9915), *Bos taurus* (cattle, NCBITaxon:9913) and *Bos indicus × Bos taurus* (hybrid cattle, NCBITaxon:30522), and *Bos grunniens* (yak, NCBITaxon:30521) following the acceptance of the animal science and veterinary communities.^10,15,16^ Since breeds are identified as distinctive groups within a family, genus or species, breeds are represented in the ontology as a subclass of these, themselves represented by NCBITaxon entities. VBO is therefore integrated within the NCBITaxon hierarchy (Figure 1B and 2).

Each term in VBO is identified by a unique and permanent ID (Table 1). Breeds from different species often share the same name, for example, “Abyssinian” is the name for a breed of horse, cat, and donkey. In addition, some breeds are commonly called by names that can represent other types of entities. For example “Cyprus” is used to refer to the name for a breed of cat, cattle, donkey, and goat but also to the country “Cyprus” (see section “Breeds originating from DAD-IS”). To ensure that all term names were unique, we created term labels by concatenating the breed’s most common name and their species common name, following the format: ‘Most common name (Species)’, in which Most common name and Species are the English language names (e.g. ‘Cyprus (Cat)’). Distinguishable sub-breeds, strains, or varieties are also included in VBO and are related to the ontological parent breed using an *is_a* relation.^17–19^ For example, ‘Chihuahua, Long-Haired (Dog)’ (VBO:0200339) and ‘Chihuahua, Smooth-Haired (Dog)’ (VBO:0200340) are subclasses of ‘Chihuahua (Dog)’ (VBO:0200338, Figure 1B).

**Figure 3:**
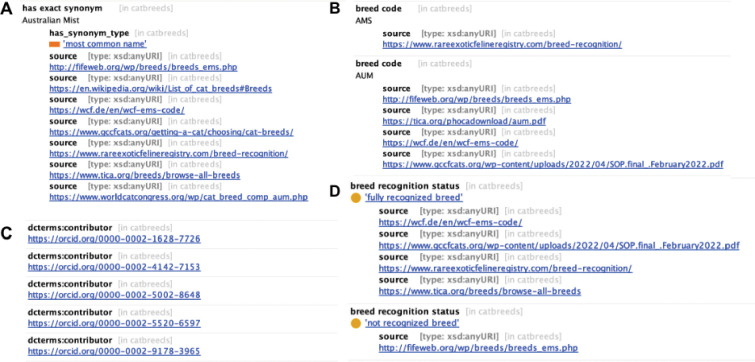
Examples of metadata for ‘Australian Mist (Cat)’ (VBO:0100034). **(A)** “has exact synonym”, “Australian Mist”, with the synonym type “most common name” indicate the name most often used to refer to the VBO term. The provenance for this information is shown as “source” annotations. **(B)** Two “breed codes” are used to refer to this VBO term: AMD and ALM. The corresponding “source” indicates which organization uses each code. **(C)** contributors who participated in the creation of this term are recorded as metadata using ORCID. **(D)** Recognition by registration bodies is recorded in “breed recognition status”. Provenance for this metadata is also recorded as “source”.

Term metadata and provenance are provided for each VBO term. Metadata fields, definitions, and examples are provided in the VBO documentation.^9^ Required term metadata include the most common name (a synonym tagged to indicate the name by which a breed is most commonly referred to), source (indicating provenance of the information), and contributor (Open Researcher and Contributor ID (ORCID)^20^ of curators and experts who contributed to the creation/revision of a VBO term) (Figure 3).

Additional metadata such as other synonym(s), database cross reference, breed codes, recognition status, domestication status, extinction status, and description of origin are included when available. Information from different sources might be discordant, for example the breed recognition by registration bodies. We chose to not be the arbitrator of breed information, and instead record all information, relying on provenance (ie. source annotations) to guide users. For example the ‘Australian Mist (Cat)’ (VBO:0100034) is a ‘fully recognized breed’ of the Governing Council of the Cat Fancy (GCCF), Rare and Exotic Feline Registry (REFR), The International Cat Association (TICA) and the World Cat Federation (WCF) and a ‘not recognized breed’ of the Fédération Internationale Féline (FIFe) (Figure 3).

### Breed Foundation Stock

**Figure 4:**
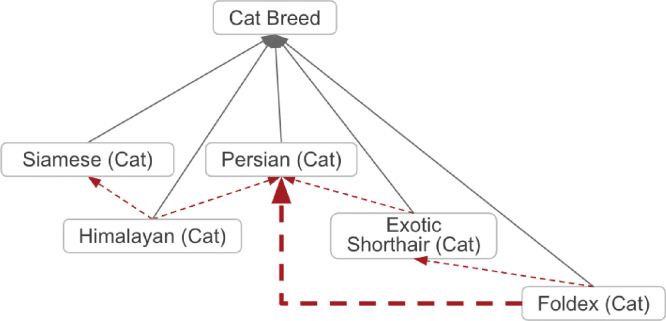
Breeds are related to their progenitors via the *has_foundation_stock* relation. ‘Himalayan (Cat)’ has progenitors *‘*Siamese (Cat)’ and ‘Persian (Cat)’; therefore this breed is related to them via the *has_foundation_stock* relation. Due to the transitive property of the *has_foundation_stock* relation, terms inherit the *has_foundation_stock* from their progenitor(s). For example, ‘Foldex (Cat)’ *has_foundation_stock ‘*Exotic Shorthair (Cat)’, which itself *has_foundation_stock* ‘Persian (Cat)’. Therefore, it can be inferred that ‘Foldex (Cat)’ *has_foundation_stock* ‘Persian (Cat)’ (thick hashed arrow). Full arrows represent *is_a* relation; hashed arrows represent *has_foundation_stock* relation. Some relations and VBO terms are not displayed in this figure for clarity. All VBO IDs are reported in Table 2.

Breeds are sometimes created by crossing other breeds whose traits and/or pedigrees are desirable. For example ‘Himalayan (Cat)’ (VBO:0100117) was created from a cross of individuals from ‘Siamese (Cat)’ (VBO:0100221) and ‘Persian (Cat)’ (VBO:0100188) (Figure 4). These animals that are the progenitors, or foundation, of a breed are called “foundation stock.”^21^ They provide part of the underlying genetic base for a new distinct population. VBO provides information about breeds’ foundation stock by using the *has_foundation_stock* relation. This relation is defined as “a relation between two distinct material entities (breeds or species), a descendant entity and an ancestor entity, in which the descendant entity is the result of mating, manipulation, or geographical or cultural isolation of the ancestor entity, therefore inheriting some of the ancestor’s genetic material.” It should be noted that foundation stock could be one or more other breeds.

### Breeds originating from DAD-IS

FAO compiles and maintains a list of breeds reported by country-nominated National Coordinators from 182 countries. The goal of this DAD-IS breed list is the management of animal genetic resources, focusing on diversity of livestock breeds on national, regional and global levels including the status of breeds regarding their risk of extinction. DAD-IS includes specific information related to its goals^10^, and therefore, the corresponding breeds in VBO have unique associated metadata and semantic information representing this information, such as domestication status and extinction status.

Most breeds in DAD-IS represent breeds that “exist in a specific country” as reported by National Coordinators.^22^ This concept, specific to DAD-IS, is represented in VBO using the ‘*located_in* some Country’ axiom indicating the country where the breed was reported, using a Wikidata entry.^23^ In addition, to ensure term label uniqueness, the naming conventions for these breeds follow the format: ‘Most common name, Country (Species)’, in which Country and Species are the English language names (Figure 2).

It is important to note that this concept of “breed that exists in a specific country” is unique to DAD-IS and its goals. While VBO users should be aware of this concept, it will rarely be used in other contexts.

### Community work

Though maintained by the Monarch Initiative^24^, VBO is a community resource that involves the participation of the community at large: anyone can request changes or new breeds to be added to the ontology through the GitHub Issue Tracker (https://github.com/monarch-initiative/vertebrate-breed-ontology/issues).

## Discussion:

VBO is a unique, open, community-driven ontology for vertebrate animal breeds, covering a broad scope of animals including livestock and companion animals, and encompassing all breeds as defined by and in the context of international organizations and communities. Ontology modeling decisions were made based on use cases. However, these have a few consequences that we address in the Supplemental Discussion. VBO is a standard for breed terms, which supports data disambiguation and integration. Its hierarchical classification of concepts, and defined relationships between concepts allow computational logical reasoning^25,26^ which can be leveraged in predictive tools. For instance, VBO supports the construction of veterinary clinical decision assistance tools that provide information about disease susceptibility in breeds, and precision medicine tools to identify optimal treatments for individual animals. In addition, VBO can be leveraged for cross-species translational research and work in the field of conservation medicine.^27^

The same breed can be referenced using different names across veterinary databases and scientific reports, since no universal standard has yet been adopted. Using VBO ID in these databases and reports disambiguates breed-related data.^28,29^ For example, the information in Online Mendelian Inheritance in Animals (OMIA) has been rendered more interoperable by using VBO terms to specify breeds in which a trait or disorder and/or a likely causal variant has been documented.^30^

Disease prediction tools are augmented by leveraging the computational logical reasoning of VBO, for example, in the context of disease susceptibility in specific breeds. For example, it has been shown that Persian cats are more susceptible to developing autosomal dominant polycystic kidney (ADPKD) disease, due to a variant in the PKD1 gene.^31–33^ The axioms in VBO indicate that ‘Persian (Cat)’ (VBO:0100188) is a foundation stock for ‘Exotic Shorthair (Cat)’ (VBO:0100096), which itself is a foundation stock for ‘Foldex (Cat)’ (VBO:0100099) (Figure 3). Based on these relationships, one could theorize that ‘Exotic Shorthair (Cat)’ and ‘Foldex (Cat)’ could also be more susceptible to ADPKD. This has indeed been reported to be the case, and it has also been shown that a variant in PKD1 is associated with this susceptibility, as in ‘Persian (Cat)’.^31–33^ Similar predictions could guide disease predictions and treatment discovery that might be appropriate for some breeds but not others of the same species.

We relied on external sources, such as international organizations, communities, and experts, to determine whether a term should be added to VBO. However, these external sources have specific purposes (e.g breed competitions, breed diversity), there are often disagreements on whether a group of animals should be recognized as a breed. For example, ‘Plott Hound (Dog)’ (VBO:0201023) is an American Kennel Club (AKC) recognized breed, but is not included in the breed list of the Fédération Cynologique Internationale (FCI). Similarly, VeNom^34^ lists ‘Labradoodle (Dog)’ (VBO:0200798) as a breed although it is not included in breed lists of canine registration bodies such as the AKC, FCI, and United Kennel Club. We took an inclusive approach and created a VBO term when “any” sources considered a breed, making VBO relevant to a broad range of use cases. We trust that, by recording the provenance of all pieces of information, users will be able to decide whether or not to include a particular VBO term for their application.

Many animal breeds have been historically based on conformation standards including structure and appearance (e.g. coat color, hair length, size), as demonstrated by reports and text descriptions in international breed organizations and breed references.^35^ Advancement in genetics brings new breed information and in some cases questions the validity of the ancestry of these breeds.^28,36^ In addition, the discovery of genetic components associated with trait aspects (e.g. variations determining cat tail length ^37^ and the effect of genotype on performance in racing ‘Standardbred (Horse)’ (VBO:0000899) and ‘Scandinavian Coldblood Trotter (Horse)’ (VBO:0017173)^38^) are changing how breeds are defined and how individuals are selected for breeding. This disparity between breeds defined by traits versus genetics can have a big impact on the veterinary data. For example, treatment efficacy might be affected by genetic factors. Therefore, predicting treatment efficacy between breeds would be more accurate if breeds are related to each other based on genetics, and not based on traits. The introduction of relationships and classifications in VBO to specify how breeds are related (i.e. genetically versus phenotypically) will augment VBO’s potential.

Veterinary EHR is the ideal data source where VBO should be implemented, as it directly interacts with clinical decision-support tools. An achievable first step would be annotating research artifacts, including journal articles and datasets with VBO IDs. This would aid in collating studies performed in animals of the same breed for the purpose of data integration in systematic reviews and meta-analyses, helping to overcome the problem that prospective veterinary studies are performed on small numbers of animals.^39^

VBO is built by the community for the community and is a blueprint and first step towards achieving data harmonization in veterinary medicine.

## Supplementary Material

Supplement 1

## Figures and Tables

**Table 1:** Minimum Information for the Reporting of an Ontology (MIRO) for VBO. This table was created based on the MIRO guidelines as described in Matentzoglu et al.^14^

A. The basics	
A.1 Ontology name	Vertebrate Breed Ontology (VBO) Version in this manuscript: v2024–05-22
A.2 Ontology owner	Monarch Initiative (https://monarchinitiative.org)
A.3 Ontology license	CC-BY 4.0
A.4 Ontology URL	http://purl.obolibrary.org/obo/vbo.owl
A.5 Ontology repository	https://github.com/monarch-initiative/vertebrate-breed-ontology
A.6 Methodological framework	Breed lists were collected from international breed organizations and communities. These lists were manually curated and integrated such that the same breed concept and its information was represented by a single VBO term. Term classification based on species (NCBITaxon) was also done manually.
**B. Motivation**	
B.1 Need	Single source for breed name and related information, representing a broad range of species and breeds, open access, and including provenance for the information.
B.2 Competition	Livestock Breed Ontology (LBO) is a resource for livestock breed. LBO is, however, limited to livestock breeds, and does not include companion animals (such as cat and dog breeds). In addition, many new livestock breeds (e.g. some breeds reported in DAD-IS) are also out of scope in VBO.
B.3 Target audience	- Databases, such as OMIA. - Any sources containing breed information; for example veterinary EHR - Publications, in order to disambiguate breeds and enable data curation and integration.
**C. Scope, requirements, development community (SRD)**	
C.1 Scope and coverage	All vertebrate breeds, including sub-breeds, varieties, strains, etc
C.2 Development community	The content of the ontology was initially created based on international breed organization and communities lists. Additional VBO terms and breed information are driven by user requests and new available (either discovered or not yet included) breed sources.
C.3 Communication	- GitHub Issue tracker: https://github.com/monarch-initiative/vertebrate-breed-ontology/issues - Mailing list: vbo-users@tislab.org (subscribe by sending an email to: vbo-users+subscribe@tislab.org)
**D. Knowledge acquisition (KA)**	
D.1 Knowledge acquisition method	Breed lists were collected from international breed organizations and communities and manually curated, with consultation with animal experts. Review and verification happens with targetted reviews involving experts, and via user requests.
D.2 Source knowledge location	Sources where the breed knowledge was gathered can be found here: https://monarch-initiative.github.io/vertebrate-breed-ontology/general/general/
D.3 Content selection	User requests for new breeds and update to the existing ontology are given priority. While synchronization of information with the original sources, as well as the addition of new breed sources are also of high priority, these are addressed on a per available ontology editor resources basis.
**E. Ontology content**	
E.1 Knowledge Representation language	The OWL language is used as it is more expressive and allows axioms such as “*located_in* value [Wikidata ID for country]”, which are necessary for the majority of the breeds from the DAD-IS list. .obo and .json versions of the ontology are also available, however, these formats do not include all information included in the .owl format (e.g. “*located_in* some country” axioms)
E.2 Development environment	The Ontology Development Kit (ODK, https://github.com/INCATools/ontology-development-kit) was used to create and is used to maintain VBO.
E.3 Ontology metrics	As of version (v2024–05-22): - 19,413 classes - 118 Object properties - 245,368 Axioms (54,804 Logical axioms)
E.4 Incorporation of other ontologies	- NCBITaxon (though not an ontology *per se*) is at the basis of VBO - Relation Ontology (RO) is used for relationships - Core Ontology for Biology and Biomedicine (COB) is used as an upper-level ontology. These are updated before each release, which aims to be monthly.
E.5 Entity naming convention	Naming conventions for term labels follow the format: - ‘Most common name (Species)’ in which Species is the English names (e.g. ‘Chihuahua (Dog)’ (VBO:0200338)) - ‘Most common name, Country; (Species)’ in which country and species are the English names. (e.g. ‘Jersey Giant, Canada; Chicken’ (VBO:0006068)), This format is used for breeds defined by their country of existence as reported by FAO National coordinators in DAD-IS More information about naming conventions here: https://monarch-initiative.github.io/vertebrate-breed-ontology/ontologymodeling/term-labels-naming-conventions/
E.6 Identifier generation policy	http://purl.obolibrary.org/obo/VBO_ followed by 7 digits E.g. http://purl.obolibrary.org/obo/VBO_0200338
E.7 Entity metadata policy	The following metadata is required: - ID - Term label - Most common name - Source - Contributor Other metadata is optional, for example: - Synonyms - Database cross-reference - Breed code - … A full list of metadata and their descriptions can be found in our documentation: https://monarch-initiative.github.io/vertebrate-breed-ontology/ontologymodeling/metadata/
E.8 Upper ontology	Core Ontology for Biology and Biomedicine (COB)
E.9 Ontology relationships	RO relations are used. The main relations used are ***is_a***, and ***located_in*** (RO:0001025; used specifically for DAD-IS breed defined by their country of existence as reported by FAO National coordinators). Additional relations specific to breed information were created in VBO. For example: **has_foundation_stock** (VBO:0300019) https://monarch-initiative.github.io/vertebrate-breed-ontology/ontologymodeling/axioms/ We plan on submitting all VBO-created relations to RO. If in scope, and new relations are created in RO, these will replace the current VBO relations.
E.10 Axiom pattern	See documentation: https://monarch-initiative.github.io/vertebrate-breed-ontology/general/general/
E.11 Dereferencable IRI	Standard prefix: VBO
**F. Managing change**	
F.1 Sustainability plan	VBO is actively developed and maintained by the members of the Monarch Initiative and the OMIA team. Training sessions and new ontology editing tools are in the works to empower more users (who are not necessarily ontologists) to edit the ontology directly.
F.2 Entity deprecation strategy	Terms deprecation happens when: - A VBO term represents a concept that never existed (i.e. created by mistake). In this case, the term is obsoleted. - Their IDs is maintained with the annotation ‘owl:deprecated’: true. - The obsoletion reason is recorded using the term ‘domain entity does not exist’. - A link to the issue tracker discussing this obsoletion is recorded using ‘term tracker item’ (IAO:0000233) annotation - VBO term represents the same breed concept as another VBO term (i.e. concepts are duplicated). In this case, the terms are merged. - The IDs of the merged term, ie that is obsoleted, is maintained with the annotation ‘owl:deprecated’: true. - The obsoletion reason is recorded using the term ‘term merged’. - The annotation “replaced by” indicate the VBO ID of the term into which it was merged - A link to the issue tracker discussing this merge is recorded using ‘term tracker item’ (IAO:0000233) annotation
F.3 Versioning policy	We aim to release a new ontology version every month.
**G. Quality Assurance (QA)**	
G.1 Testing	We use the general QA/QC included in the ODK framework. In addition, we are working on creating new QC tests specific to VBO.
G.2 Evaluation	Currently, the ontology is evaluated by - users who use VBO in data annotations, and checking that all required VBO terms exist - Ontology developers who are reviewing the ontology by comparing it to major breed sources (e.g Mason’s World Dictionary of Livestock Breeds, Types and Varieties and Mason's World Encyclopedia of Livestock Breeds and Breeding) Evaluation using data integration and competency questions will be possible once VBO has been fully adopted by multiple databases.
G.3 Examples of use	- https://omia.org/OMIA000515/9685/ - https://omia.org/OMIA001081/9615/
G.4 Institutional endorsement	VBO has been accepted to the OBO foundry.^13^
G.5 Evidence of use	- OMIA (https://omia.org/home/) records breed information and use VBO ID to refer to breeds - Selected publications using VBO ID to refer to breeds^28,29^

**Table 2:** List of identifiers reported in this publication. Note that the category “VBO term-breed” includes sub-breed, variety, etc.

Categories	Labels	IDs	Location in the document
VBO term-breed	Aberdeen Angus, Brazil (Cattle)	VBO:0002150	Fig.2
VBO term-breed	Aberdeen Angus, Ireland (Cattle)	VBO:0002169	Fig.2
VBO term-breed	Aberdeen-Angus (Cattle)	VBO:0000090	Fig.2
VBO term-breed	American Miniature Horse (Horse)	VBO:0000896	Suppl.
VBO term-breed	Appaloosa (Horse)	VBO:0000904	Suppl.
VBO term-breed	Australian Mist (Cat)	VBO:0100034	Text, Fig.4
VBO term-breed	Beagle (Dog)	VBO:0200131	Suppl.
VBO term-breed	Chihuahua (Dog)	VBO:0200338	Fig.1
VBO term-breed	Chihuahua (Dog)	VBO:0200338	Table 1
VBO term-breed	Chihuahua, Long-Haired (Dog)	VBO:0200339	Text, Fig.1
VBO term-breed	Chihuahua, Smooth-Haired (Dog)	VBO:0200340	Text, Fig.1
VBO term-breed	Cyprus (Cat)	VBO:0100081	Text
VBO term-breed	Eastern Yak, Bhutan (Yak (domestic))	VBO:0016815	Fig.2
VBO term-breed	Exotic Shorthair (Cat)	VBO:0100096	Fig.3
VBO term-breed	Foldex (Cat)	VBO:0100099	Fig.3
VBO term-breed	Guraghe, Ethiopia (Cattle)	VBO:0004734	Fig.2
VBO term-breed	Himalayan (Cat)	VBO:0100117	Text, Fig.3
VBO term-breed	Jersey Giant, Canada; Chicken	VBO:0006068	Table 1
VBO term-breed	Knabstrupper (Horse)	VBO:0001008	Suppl. Fig.1
VBO term-breed	Labradoodle (Dog)	VBO:0200798	Text
VBO term-breed	Lakenvelder, Belgium (Cattle)	VBO:0002866	Suppl. Fig.1
VBO term-breed	Persian (Cat)	VBO:0100188	Text, Fig.3
VBO term-breed	Plott Hound (Dog)	VBO:0201023	Text
VBO term-breed	Scandinavian Coldblood Trotter	VBO:0017173	Text
VBO term-breed	Siamese (Cat)	VBO:0100221	Text, Fig.3
VBO term-breed	Standardbred (Horse)	VBO:0000899	Text
VBO term-breed	Zebu (Cattle)	VBO:0017417	Fig.2
VBO term-breed	Zebu, Australia (Cattle)	VBO:0004402	Fig.2
VBO term-breed	Zebu, Guyana (Cattle)	VBO:0004839	Fig.2
VBO term-classification	Alpaca breed	VBO:0000038	Fig.1
VBO term-classification	American bison breed	VBO:0000041	Fig.1, Fig.2
VBO term-classification	Ass breed	VBO:0400005	Fig.1
VBO term-classification	Bird breed	VBO:0400006	Fig.1, Suppl.
VBO term-classification	Bovine breed	VBO:0400019	Fig.1, Fig.2
VBO term-classification	Buffalo breed	VBO:0000068	Fig.1, Fig.2
VBO term-classification	Camel breed	VBO:0400022	Fig.1
VBO term-classification	Cat breed	VBO:0400018	Fig.1, Fig.3
VBO term-classification	Cattle breed	VBO:0400020	Text, Fig.1, Fig.2, Suppl.
VBO term-classification	Chicken breed	VBO:0400010	Suppl., Suppl. Fig.1
VBO term-classification	Deer breed	VBO:0400023	Fig.1
VBO term-classification	Dog breed	VBO:0400024	Text, Fig.1
VBO term-classification	Equid breed	VBO:0400033	Fig.1
VBO term-classification	Goat breed	VBO:0400025	Fig.1
VBO term-classification	Guanaco breed	VBO:0000882	Fig.1
VBO term-classification	Guinea pig breed	VBO:0400026	Fig.1
VBO term-classification	Horse breed	VBO:0000931	Fig.1
VBO term-classification	Llama breed	VBO:0001098	Fig.1
VBO term-classification	Partridge breed	VBO:0400038	Suppl., Suppl. Fig.1
VBO term-classification	Pheasant breed	VBO:0400037	Suppl., Suppl. Fig.1
VBO term-classification	Pig breed	VBO:0001199	Fig.1
VBO term-classification	Quail breed	VBO:0001223	Suppl., Suppl. Fig.1
VBO term-classification	Rabbit breed	VBO:0400029	Fig.1
VBO term-classification	Sheep breed	VBO:0400030	Fig.1
VBO term-classification	South American camelid breed	VBO:0400032	Fig.1
VBO term-classification	Vertebrate Breed	VBO:0400000	Text, Fig.1, Suppl.
VBO term-classification	Vicuña breed	VBO:0001721	Fig.1
Taxon	Aves	NCBITaxon:8782	Suppl.
Taxon	*Bos*	NCBITaxon:9903	Fig.2, Suppl.
Taxon	*Bos grunniens*	NCBITaxon:30521	Fig.2
Taxon	*Bos indicus*	NCBITaxon:9915	Fig.2
Taxon	*Bos indicus × Bos taurus*	NCBITaxon:30522	Text
Taxon	*Bos taurus*	NCBITaxon:9913	Fig.2
Taxon	*Bovinae*	NCBITaxon:27592	Fig.2
Taxon	*Canis lupus familiaris*	NCBITaxon:9615	Fig.1
Taxon	*Coturnix*	NCBITaxon:9090	Suppl. Fig.1
Taxon	*Gallus*	NCBITaxon:9030	Suppl. Fig.1
Taxon	*Gallus gallus*	NCBITaxon:9031	Suppl., Suppl. Fig.1
Taxon	*Perdicinae*	NCBITaxon:466544	Suppl. Fig.1
Taxon	*Phasianinae*	NCBITaxon:9072	Suppl., Suppl. Fig.1
Taxon	Vertebrata <vertebrates>	NCBITaxon:7742	Fig.1
Breed status	domestication status	VBO:0300005	Text
Breed status	extinction status	VBO:0300009	Text
Breed status	fully recognized breed	VBO:0300002	Text
Breed status	not recognized breed	VBO:0300004	Text
relation	*has_foundation_stock*	VBO:0300019	Text, Table 1
relation	*located_in*	RO:0001025	Table 1

## References

[1] Chon E, Hendricks W, White M, Rodrigues L, Haworth D, Post G. Precision Medicine in Veterinary Science. Vet Clin North Am Small Anim Pract. 2024 May;54(3):501–21. 38212188 10.1016/j.cvsm.2023.12.006

[2] Mealey KL, Martinez SE, Villarino NF, Court MH. Personalized medicine: going to the dogs? Hum Genet. 2019 May;138(5):467–81. 31032534 10.1007/s00439-019-02020-w

[3] Vaidhya A, Ghildiyal K, Rajawat D, Nayak SS, Parida S, Panigrahi M. Relevance of pharmacogenetics and pharmacogenomics in veterinary clinical practice: A review. Anim Genet. 2024 Feb;55(1):3–19. 37990577 10.1111/age.13376

[4] Thamm DH, Gustafson DL. Drug dose and drug choice: Optimizing medical therapy for veterinary cancer. Vet Comp Oncol. 2020 Jun;18(2):143–51. 31487110 10.1111/vco.12537

[5] Nosalova N, Huniadi M, Horňáková Ľ, Valenčáková A, Horňák S, Nagoos K, Canine Mammary Tumors: Classification, Biomarkers, Traditional and Personalized Therapies. Int J Mol Sci [Internet]. 2024 Mar 1;25(5). Available from: 10.3390/ijms25052891 PMC10931591 38474142

[6] Klopfleisch R. Personalised medicine in veterinary oncology: one to cure just one. Vet J. 2015 Aug;205(2):128–35. 25641551 10.1016/j.tvjl.2015.01.004

[7] Buckley RM, Lyons LA. Precision/Genomic Medicine for Domestic Cats. Vet Clin North Am Small Anim Pract. 2020 Sep;50(5):983–90. 32653264 10.1016/j.cvsm.2020.05.005

[8] Callahan TJ, Stefanski AL, Wyrwa JM, Zeng C, Ostropolets A, Banda JM, Ontologizing health systems data at scale: making translational discovery a reality. NPJ Digit Med. 2023 May 19;6(1):89. 37208468 10.1038/s41746-023-00830-x PMC10196319

[9] VBO general information - Vertebrate Breed Ontology [Internet]. [cited 2024 May 28]. Available from: https://monarch-initiative.github.io/vertebrate-breed-ontology/general/general/

[10] Domestic Animal Diversity Information System (DAD-IS) [Internet]. [cited 2024 May 28]. Available from: https://www.fao.org/dad-is/en/

[11] Matentzoglu N, Goutte-Gattat D, Tan SZK, Balhoff JP, Carbon S, Caron AR, Ontology Development Kit: a toolkit for building, maintaining and standardizing biomedical ontologies. Database [Internet]. 2022 Oct 8;2022. Available from: 10.1093/database/baac087 PMC9547537 36208225

[12] Jackson RC, Balhoff JP, Douglass E, Harris NL, Mungall CJ, Overton JA. ROBOT: A Tool for Automating Ontology Workflows. BMC Bioinformatics. 2019 Jul 29;20(1):407. 31357927 10.1186/s12859-019-3002-3 PMC6664714

[13] Jackson R, Matentzoglu N, Overton JA, Vita R, Balhoff JP, Buttigieg PL, OBO Foundry in 2021: operationalizing open data principles to evaluate ontologies. Database [Internet]. 2021 Oct 26;2021. Available from: 10.1093/database/baab069 PMC8546234 34697637

[14] Matentzoglu N, Malone J, Mungall C, Stevens R. MIRO: guidelines for minimum information for the reporting of an ontology. J Biomed Semantics. 2018 Jan 18;9(1):6. 29347969 10.1186/s13326-017-0172-7 PMC5774126

[15] Zhang R. Interspecies Hybridization between Yak, Bos taurus and Bos indicus and Reproduction of the Hybrids [Internet]. IVIS. 2000 [cited 2024 May 24]. Available from: https://www.ivis.org/library/recent-advances-yak-reproduction/interspecies-hybridization-between-yak-bos-taurus-and-bos

[16] LBO Project [Internet]. [cited 2024 May 28]. Available from: https://www.animalgenome.org/bioinfo/projects/lbo/

[17] FCI breeds nomenclature [Internet]. [cited 2024 May 28]. Available from: https://www.fci.be/en/Nomenclature/varietes.aspx

[18] Inc American Poultry Association. American Standard of Perfection 2010. 2010th ed. American Poultry Association; 1894.

[19] González Ariza A, Nogales Baena S, Lupi TM, Arando Arbulu A, Navas González FJ, León Jurado JM, Characterisation of biological growth curves of different varieties of an endangered native hen breed kept under free range conditions. Ital J Anim Sci. 2021 Jan 1;20(1):806–13.

[20] ORCID [Internet]. [cited 2024 Jun 2]. Available from: https://orcid.org/

[21] Lush JL. Animal Breeding Plans. Iowa State College Press; 1945. 443 p.

[22] The Second Report on the State of the World’s Animal Genetic Resources for Food and Agriculture [Internet]. [cited 2024 May 28]. Available from: https://www.fao.org/policy-support/tools-and-publications/resources-details/en/c/435207/

[23] Wikidata [Internet]. [cited 2024 Jun 1]. Available from: https://www.wikidata.org/wiki/Wikidata:Main_Page

[24] Putman TE, Schaper K, Matentzoglu N, Rubinetti VP, Alquaddoomi FS, Cox C, The Monarch Initiative in 2024: an analytic platform integrating phenotypes, genes and diseases across species. Nucleic Acids Res. 2024 Jan 5;52(D1):D938–49. 38000386 10.1093/nar/gkad1082 PMC10767791

[25] Haendel MA, Chute CG, Robinson PN. Classification, Ontology, and Precision Medicine. N Engl J Med. 2018 Oct 11;379(15):1452–62. 30304648 10.1056/NEJMra1615014 PMC6503847

[26] Chute CG. Clinical classification and terminology: some history and current observations. J Am Med Inform Assoc. 2000 May-Jun;7(3):298–303. 10833167 10.1136/jamia.2000.0070298 PMC61433

[27] Gudra D, Valdovska A, Jonkus D, Kairisa D, Galina D, Ustinova M, Genetic characterization of the Latvian local goat breed and genetic traits associated with somatic cell count. Animal. 2024 May;18(5):101154. 38703755 10.1016/j.animal.2024.101154

[28] Rando HM, Graim K, Hampikian G, Greene CS. Many direct-to-consumer canine genetic tests can identify the breed of purebred dogs. J Am Vet Med Assoc. 2024 Feb 27;1–8. 10.2460/javma.23.07.0372 38417257

[29] Esdaile E, Knickelbein KE, Donnelly CG, Ferneding M, Motta MJ, Story BD, Additional evidence supports GRM6 p.Thr178Met as a cause of congenital stationary night blindness in three horse breeds. Vet Ophthalmol [Internet]. 2023 Oct 10; Available from: 10.1111/vop.13151 PMC13267762 37815029

[30] Nicholas F, Tammen I, Hub SI. Online Mendelian Inheritance in Animals (OMIA) [Internet]. The University of Sydney; 1995 [cited 2024 Jun 1]. Available from: https://ses.library.usyd.edu.au/handle/2123/31190

[31] Lyons LA, Biller DS, Erdman CA, Lipinski MJ, Young AE, Roe BA, Feline polycystic kidney disease mutation identified in PKD1. J Am Soc Nephrol. 2004 Oct;15(10):2548–55. 15466259 10.1097/01.ASN.0000141776.38527.BB

[32] Shitamori F, Nonogaki A, Motegi T, Matsumoto Y, Sakamoto M, Tanizawa Y, Large-scale epidemiological study on feline autosomal dominant polycystic kidney disease and identification of novel PKD1 gene variants. J Feline Med Surg. 2023 Jul;25(7):1098612X231185393. 10.1177/1098612X231185393 PMC10812055 37489504

[33] Polycystic Kidney Disease (PKD1) [Internet]. [cited 2024 May 23]. Available from: https://vgl.ucdavis.edu/test/pkd1-cat

[34] VeNom Coding [Internet]. [cited 2024 May 29]. Available from: https://venomcoding.org/

[35] Mason’s world encyclopedia of livestock breeds and breeding. Volume 1 and Volume 2 [Internet]. CABI Books. [cited 2024 Mar 11]. Available from: https://www.cabidigitallibrary.org/doi/book/10.1079/9781845934668.0000

[36] Cosgrove EJ, Sadeghi R, Schlamp F, Holl HM, Moradi-Shahrbabak M, Miraei-Ashtiani SR, Genome Diversity and the Origin of the Arabian Horse. Sci Rep. 2020 Jun 16;10(1):9702. 32546689 10.1038/s41598-020-66232-1 PMC7298027

[37] Buckingham KJ, McMillin MJ, Brassil MM, Shively KM, Magnaye KM, Cortes A, Multiple mutant T alleles cause haploinsufficiency of Brachyury and short tails in Manx cats. Mamm Genome. 2013 Oct;24(9–10):400–8. 23949773 10.1007/s00335-013-9471-1 PMC3848309

[38] Jäderkvist K, Andersson LS, Johansson AM, Árnason T, Mikko S, Eriksson S, The DMRT3 “Gait keeper” mutation affects performance of Nordic and Standardbred trotters. J Anim Sci. 2014 Oct;92(10):4279–86. 25085403 10.2527/jas.2014-7803

[39] Wareham KJ, Hyde RM, Grindlay D, Brennan ML, Dean RS. Sample size and number of outcome measures of veterinary randomised controlled trials of pharmaceutical interventions funded by different sources, a cross-sectional study. BMC Vet Res. 2017 Oct 4;13(1):295. 28978314 10.1186/s12917-017-1207-0 PMC5628436

[40] Vasilevsky NA, Matentzoglu NA, Toro S, Flack JE IV, Hegde H, Unni DR, Mondo: Unifying diseases for the world, by the world [Internet]. bioRxiv. 2022. Available from: 10.1101/2022.04.13.22273750v3.abstract

[41] Bellone RR, Holl H, Setaluri V, Devi S, Maddodi N, Archer S, Evidence for a retroviral insertion in TRPM1 as the cause of congenital stationary night blindness and leopard complex spotting in the horse. PLoS One. 2013 Oct 22;8(10):e78280. 24167615 10.1371/journal.pone.0078280 PMC3805535

[42] Porter V. Mason’s World Dictionary of Livestock Breeds, Types and Varieties. 6th ed. CABI; 2020. 360 p.

[43] Caufield JH, Hegde H, Emonet V, Harris NL, Joachimiak MP, Matentzoglu N, Structured Prompt Interrogation and Recursive Extraction of Semantics (SPIRES): a method for populating knowledge bases using zero-shot learning. Bioinformatics [Internet]. 2024 Mar 4;40(3). Available from: 10.1093/bioinformatics/btae104 PMC10924283 38383067

