# Supplementary material for: The Vertebrate Breed Ontology: Towards effective breed data standardization
Source: ArXiv. 2025 Jan 24:arXiv:2406.02623v2. Originally published 2024 Jun 3. Preprint. [Version 2] (PMC11177956)
Supplement: Supplement 1 [file NIHPP2406.02623V2-supplement-1.pdf]

Supplementary Table: Identifiers reported in this publication.

Categories	Labels	IDs	Location in the document
VBO term-breed	Aberdeen Angus, Brazil (Cattle)	VBO:0002150	Fig.2
VBO term-breed	Aberdeen Angus, Ireland (Cattle)	VBO:0002169	Fig.2
VBO term-breed	Aberdeen-Angus (Cattle)	VBO:0000090	Fig.2
VBO term-breed	American Miniature Horse (Horse)	VBO:0000896	Text
VBO term-breed	Appaloosa (Horse)	VBO:0000904	Text
VBO term-breed	Australian Mist (Cat)	VBO:0100034	Text, Fig.4
VBO term-breed	Beagle (Dog)	VBO:0200131	Text
VBO term-breed	Chihuahua (Dog)	VBO:0200338	Fig.1
VBO term-breed	Chihuahua (Dog)	VBO:0200338	Table 1
VBO term-breed	Chihuahua, Long-Haired (Dog)	VBO:0200339	Text, Fig.1
VBO term-breed	Chihuahua, Smooth-Haired (Dog)	VBO:0200340	Text, Fig.1
VBO term-breed	Cyprus (Cat)	VBO:0100081	Text
VBO term-breed	Eastern Yak, Bhutan (Yak (domestic))	VBO:0016815	Fig.2
VBO term-breed	Exotic Shorthair (Cat)	VBO:0100096	Fig.3
VBO term-breed	Foldex (Cat)	VBO:0100099	Fig.3
VBO term-breed	Guraghe, Ethiopia (Cattle)	VBO:0004734	Fig.2
VBO term-breed	Himalayan (Cat)	VBO:0100117	Text, Fig.3
VBO term-breed	Jersey Giant, Canada; Chicken	VBO:0006068	Table 1
VBO term-breed	Knabstrupper (Horse)	VBO:0001008	Fig.5
VBO term-breed	Labradoodle (Dog)	VBO:0200798	Text
VBO term-breed	Lakenvelder, Belgium (Cattle)	VBO:0002866	Fig.5

VBO term-breed	Persian (Cat)	VBO:0100188	Text, Fig.3
VBO term-breed	Plott Hound (Dog)	VBO:0201023	Text
VBO term-breed	Scandinavian Coldblood Trotter (Horse)	VBO:0017173	Text
VBO term-breed	Siamese (Cat)	VBO:0100221	Text, Fig.3
VBO term-breed	Standardbred (Horse)	VBO:0000899	Text
VBO term-breed	Zebu (Cattle)	VBO:0017417	Fig.2
VBO term-breed	Zebu, Australia (Cattle)	VBO:0004402	Fig.2
VBO term-breed	Zebu, Guyana (Cattle)	VBO:0004839	Fig.2
VBO term-classification	Alpaca breed	VBO:0000038	Fig.1
VBO term-classification	American bison breed	VBO:0000041	Fig.1, Fig.2
VBO term-classification	Ass breed	VBO:0400005	Fig.1
VBO term-classification	Bird breed	VBO:0400006	Text, Fig.1
VBO term-classification	Bovine breed	VBO:0400019	Fig.1, Fig.2
VBO term-classification	Buffalo breed	VBO:0000068	Fig.1, Fig.2
VBO term-classification	Camel breed	VBO:0400022	Fig.1
VBO term-classification	Cat breed	VBO:0400018	Fig.1, Fig.3
VBO term-classification	Cattle breed	VBO:0400020	Text, Fig.1, Fig.2
VBO term-classification	Chicken breed	VBO:0400010	Text, Fig. 5
VBO term-classification	Deer breed	VBO:0400023	Fig.1
VBO term-classification	Dog breed	VBO:0400024	Text, Fig.1
VBO term-classification	Equid breed	VBO:0400033	Fig.1
VBO term-classification	Goat breed	VBO:0400025	Fig.1
VBO term-classification	Guanaco breed	VBO:0000882	Fig.1
VBO term-classification	Guinea pig breed	VBO:0400026	Fig.1

VBO term-classification	Horse breed	VBO:0000931	Fig.1
VBO term-classification	Llama breed	VBO:0001098	Fig.1
VBO term-classification	Partridge breed	VBO:0400038	Text, Fig. 5
VBO term-classification	Pheasant breed	VBO:0400037	Text, Fig. 5
VBO term-classification	Pig breed	VBO:0001199	Fig.1
VBO term-classification	Quail breed	VBO:0001223	Text, Fig. 5
VBO term-classification	Rabbit breed	VBO:0400029	Fig.1
VBO term-classification	Sheep breed	VBO:0400030	Fig.1
VBO term-classification	South American camelid breed	VBO:0400032	Fig.1
VBO term-classification	Vertebrate Breed	VBO:0400000	Text, Fig.1
VBO term-classification	Vicuña breed	VBO:0001721	Fig.1
Taxon	Aves	NCBITaxon:8782	Text
Taxon	*Bos*	NCBITaxon:9903	Text, Fig.2
Taxon	*Bos grunniens*	NCBITaxon:30521	Fig.2
Taxon	*Bos indicus*	NCBITaxon:9915	Fig.2
Taxon	*Bos indicus × Bos taurus*	NCBITaxon:30522	Text
Taxon	*Bos taurus*	NCBITaxon:9913	Fig.2
Taxon	*Bovinae*	NCBITaxon:27592	Fig.2
Taxon	*Canis lupus familiaris*	NCBITaxon:9615	Fig.1
Taxon	*Coturnix*	NCBITaxon:9090	Fig.5
Taxon	*Gallus*	NCBITaxon:9030	Fig.5
Taxon	*Gallus gallus*	NCBITaxon:9031	Text, Fig. 5
Taxon	*Perdicinae*	NCBITaxon:466544	Fig.5
Taxon	*Phasianinae*	NCBITaxon:9072	Text, Fig. 5

Taxon	Vertebrata <vertebrates>	NCBITaxon:7742	Fig.1
Gene	PKD1, polycystin 1, transient receptor potential channel interacting, Felis catus (domestic cat)	NCBIGene:100144606	Text
Disease	congenital stationary night blindness, TRPM1-related, horse	MONDO:1011255	Text
Disease	polycystic kidney disease, domestic cat	MONDO:1011054	Text
Breed status	domestication status	VBO:0300005	Text
Breed status	extinction status	VBO:0300009	Text
Breed status	fully recognized breed	VBO:0300002	Text
Breed status	not recognized breed	VBO:0300004	Text
relation	*breed reported in geographic location*	VBO:0300020	Text, Table 1
relation	*has foundation stock*	VBO:0300019	Text, Table 1

Note that the category “VBO term-breed” includes sub-breed and variety.
