# Supplementary material for: The Vertebrate Breed Ontology: Towards Effective Breed Data Standardization
Source: ArXiv. 2024 Jun 3:arXiv:2406.02623v1. Preprint. [Version 1] (PMC11177956)
Supplement: Supplementary file 1 [file NIHPP2406.02623v1-supplement-1.pdf]

Supplemental data

Supplemental discussion

VBO was created by reviewing and curating breed lists from international organizations, communities, and experts. Ontology modeling decisions were made based on use cases. These have a few consequences that we address here.

Based on our broad definition of vertebrate breed, “a group of animals that share specific characteristics that distinguish it from other organisms of the same species (...)”, breeds were created as subclasses of species, which are represented by the NCBITaxon entities; hence, we integrated VBO terms within the NCBITaxon hierarchy using the *is_a* relation. This has two main advantages: first, since NCBITaxon is an already accepted standard in ontologies and databases, it bridges VBO to other ontologies which use NCBITaxon in axioms (e.g. Mondo Disease Ontology[40]). Second, through term axioms and logical definitions, we can leverage the species classification from NCBITaxon to autoclassify VBO terms. For example, by defining ‘Cattle breed' as ‘‘vertebrate breed’ and ‘*Bos*’, all VBO terms which are a subclass of the NCBITaxon are automatically classified as ‘Cattle breed’ (Figure 2). One should, however, be aware that the NCBITaxon hierarchy is motivated by evolution and therefore, some classification might be unintuitive to some users. For example, ‘Chicken breed’ (VBO:0400010) is defined as a “vertebrate breed of the taxon *Gallus gallus*”. In NCBITaxon, ‘*Gallus gallus*’ (NCBITaxon:9031) is a subclass of ‘*Phasianinae*’ (NCBITaxon:9072). Therefore, ‘Chicken breed’ is classified as a subclass of ‘Pheasant breed’ (VBO:0400037) since ‘Pheasant breed’ is defined as “vertebrate breed of the taxon *Phasianinae*” (Supplemental Figure 1A). Similarly, ‘Quail breed’ (VBO:0001223) is a subclass of ‘Partridge breed’ (VBO:0400038)(Supplemental Figure 1B). While we recognize that this classification is not intuitive to livestock experts, we trust that the use of synonyms and search engines will support the identification of VBO terms in these breed groups.

The inclusion of species (and country when applicable) in VBO term labels makes these labels unwieldy and cumbersome, and somewhat represents the ontology hierarchy and axioms, which is not regular ontology practice. We investigated several avenues to create more user-friendly term labels. However, since the same common names can refer to breeds of different species, and no other specific breed attribute could be consistently identified, including species in the term label naming conventions was the most reliable solution to ensure term label uniqueness.

It should be noted that the VBO terms represent a group of animals, and not individual animals. Using VBO in an annotation, for example “Snoopy” is a ‘Beagle (Dog)’ (VBO:0200131), means that “Snoopy” belongs to a group of animals called ‘Beagle (Dog)’, and shares the same characteristics as this group of animals. This is a crucial point to understand, specifically when working with the DAD-IS breed list in which each concept represents “a breed that was reported by National Coordinators to exist in a specific country”[10], and not the actual location of an individual animal. For example a cow of breed ‘Lakenvelder, Belgium (Cattle)’ (VBO:0002866) represents a cow that belongs to a breed of Lakenvelder reported to exist in Belgium. If this cow

is transported across country boundaries, and physically is in France, this cow would still be considered a member of the breed that was reported to exist in Belgium.

A wealth of breed information exists and is relevant to breeding management and veterinary care. Breeds are often defined by their phenotypic features or traits which are often associated with genetic components. For example, horse breeds with leopard complex spotting coat color pattern such as ‘Appaloosa (Horse)’ (VBO:0000904), ‘American Miniature Horse (Horse)’ (VBO:0000896) and ‘Knabstrupper (Horse)’ (VBO:0001008) are susceptible to congenital stationary night blindness, a disease that occurs in horses with leopard complex spotting coat color pattern.[41] Therefore, any knowledge related to this disease (for example, susceptibility) could be predicted to be applicable to all breeds sharing the leopard complex spotting coat color pattern. We are planning on including phenotype/trait information in future versions of VBO.

In addition, communities often classify breeds based on their usage, such as “use for meat production”, “used for milk production”, “draft horse”, “sighthound”, etc. Such classification would also be relevant to add to future VBO versions.

The majority of the VBO content was created by manually reviewing existing lists of breeds, and classifying them according to their species. However, the number of breeds and the wealth of available information is massive. In addition, the majority of available information is in free text format in databases, publication, health records, or books.[35,42] Therefore, manual review used to create VBO is neither sustainable, nor scalable. We will leverage the advances of LLM in ontology curation tools[43] to populate VBO with relevant information.

Supplemental figure

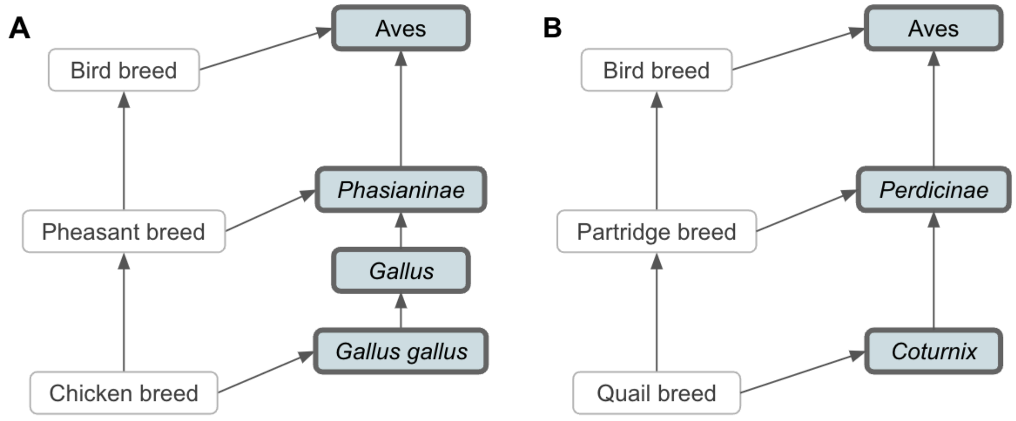

Supplemental Figure 1: NCBITaxon is leveraged to autoclassify VBO terms.
(A) NCBITaxon classifies ‘*Gallus gallus*’ is a subclass of *‘Phasianinae’.* Since ‘Chicken breed’ is a subclass of ‘*Gallus gallus*’, and ‘Pheasant breed’ is a subclass of *‘Phasianinae’*, ‘Chicken breed’ is autoclassified as a subclass of ‘Pheasant breed’. **(B)** NCBITaxon classifies ‘*Coturnix*’ is a subclass of ‘*Perdicinae’.* Since ‘Quail breed’ is a subclass of ‘*Coturnix*’, and ‘Partridge breed’ is a subclass of *‘Perdicinae’*, ‘Quail breed’ is autoclassified as a subclass of ‘Partridge breed’.
Terms from the NCBITaxon hierarchy are shown in thick border boxes. Arrows represent *is_a* relation. Some relations, NCBITaxon and VBO terms are not displayed in this figure for clarity. All VBO and NCBITaxon IDs are reported in Table 2.
